# Ethanol Gas Sensitivity Sensor Based on Roughened POF Taper of Modified Polypyrrole Films

**DOI:** 10.3390/s20040989

**Published:** 2020-02-12

**Authors:** Wenyi Liu, Yanjun Hu, Yulong Hou

**Affiliations:** Science and Technology on Electronic Test & Measurement Laboratory, North University of China, Taiyuan 030051, China

**Keywords:** polypyrrole films, roughened POF taper, ethanol gas

## Abstract

The three polypyrrole (PPy) films with different mixture ratios, namely PPy1, PPy2, and PPy3, were synthesized by chemical oxidation with pyrrole and ferric chloride (FeCl_3_). The roughened plastic optical fiber (POF) taper assembled PPy films (POF-PPy1, POF-PPy2, and POF-PPy3) were facilely prepared and bent U shape structure for testing ethanol gas at room temperature. The morphologies of the PPy films and the roughened POF taper were studied using electron microscopy. The effect of the three PPy films on the gas response was investigated and the results showed that the POF-PPy2 exhibited a high sensitivity of 5.08 × 10^−5^ dB/ppm. The detection limit of the sensor was 140 ppm and its response and recovery times were 5 s and 8 s, respectively. The results also showed that as the bending radius decreased, the response and recovery times gradually shortened, while the output power increased. In addition, the proposed sensor has advantages of a low cost and simple structure.

## 1. Introduction

Due to the high numerical aperture (NA), proper curvature, and secure processing, plastic optical fibers (POFs) have attracted significant attention from researchers and have been widely used in different applications such as gas detection, biomedical, and other fields [1,2,3,4]. The D-shaped [5,6], tapered [7], U-shaped [8], and twisted macro-bend coupling structures [9] have been fabricated and characterized. In the above structures, tapered POFs can strongly enhance the evanescent wave (EW) power in the cladding, increasing environmental sensitivity. The preparation of tapered POFs primarily relies on chemical etching [10] and heat-and-pull techniques [11]. An organic solvent, such as acetone that dissolves the cladding and core, can be used to form a non-cladding POF taper [12,13,14]. Due to their strong EW, the non-cladding fiber tapers have been used for evanescent absorption measurements and fluorescence excitation/collection [11]. However, the usage of chemical etching is explosive in the case of a complex configuration ratio. At the same time, the residual solvent on the fiber surface can change the properties of the fiber material and thus affect the subsequent application. The heat-and-pull technique can be used to form a cladding POF taper [15,16]. The cladding of a taper remains and the damage to the robustness is small. It is well known that different taper ratios have different effects on EW power. However, compared with the non-cladding POF taper, weak EW intensity does not respond significantly to changes in the external environment. The sandpaper is also a method of POF processing [5,6], namely, a method for forming non-cladding fiber. The adhesion of sandpaper-polished POF changes with its surface roughness [17,18]. In the present study, a roughened POF taper is processed by sandpapers and the fiber surface is in close contact with polypyrrole (PPy) films according to the adhesion. At the same time, the friction force of the roughened POF prevents the movement of the PPy film on the surface of the fiber, making the proposed sensor more stable. The rougher the POF surface, the greater the friction force. The machining method is simple and does not require expensive equipment.

The PPy has the advantages of simple preparation methods and high conductivity [19,20]. The synthesis of the PPy includes electrochemical polymerization and chemical polymerization. The electrochemical polymerization requires the electrodes and electrolytes, but using these inevitably increases the complexity and cost of the PPy preparation process. The chemical polymerization can control the PPy morphology by varying the reactant concentration and reaction time. Ferric chloride (FeCl_3_), ammonium sulfate, and ammonium persulfate were used as oxidants to prepare PPy films [21,22,23]. Machida et al. [24] studied the effects of different configuration solutions on the oxidation of FeCl_3_ and pyrrole. Their experimental results showed that when methanol was used as a solution, the conductivity of PPy was the highest. The ratio of FeCl_3_ to pyrrole of 2.33:1 is expressed as follows:

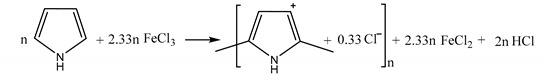
(1)

Using the configuration ratio based on the Equation (1), Qin et al. [25] synthesized a PPy film by using interfacial polymerization. Their experiments showed that the fiber modified with the PPy sensor had a high sensitivity to alcohol and the response time was tens of seconds. Zhang et al. [26] prepared a PPy film with a mass ratio of ferric chloride to pyrrole of 2.4:1. The experiments demonstrated that the sensor responded well to ammonia, ethanol, and other gases. Pirsa et al. [27] discussed the effect of the ratio of FeCl_3_ to pyrrole on the morphology and gas sensitivity of PPy films; the ratio of FeCl_3_ to pyrrole was set to 5:1, 5:2, 2:1, and 1:1, respectively. The results showed that the PPy film had the highest sensitivity to the volatile organic compounds (VOCs) when the ratio was 5:1. In this study, the effect of the reactant ratio greater than 5:1 on the thickness and gas sensitivity of PPy films was studied.

The PPy films were prepared using chemical oxidation, with the substance ratio of FeCl_3_ to pyrrole of 5:1; 15:2; and 10:1. The PPy films were modified on the surface of the POF polished with sandpaper to form three sensitive probes, namely, POF-PPy1, POF-PPy2, and POF-PPy3. The sensitivity at different bending radii was also analyzed at room temperature (RT). The experimental results showed that the POF-PPy2 had a fast response and high sensitivity. The response and recovery times were 5 s and 8 s, respectively, when the concentration of the ethanol gas was 140 ppm, and the sensitivity was 5.08 × 10^−5^ dB/ppm. When the bending radius was 3 cm and the concentration of the ethanol gas was 1400 ppm, the response and recovery times of the proposed sensor were 15 s and 17 s, respectively.

## 2. Sensing Mechanism

The V-number (normalized frequency) characterizes the number of propagation modes in optical fiber [28,29] and it is expressed as:

(2) $$V=\frac{2\pi r}{\lambda }\sqrt{n_{co}-n_{cl}}$$

where λ is the wavelength of the light source, r is the core radius, and $n_{co}$ and $n_{cl}\;$ are the refractive indexes of the core and cladding, respectively. The light of the tapered fiber is transmitted internally, resulting in the attenuated total internal reflection effect. In the tapered region, the high-order guided modes are filtered out, thus increasing the evanescent field strength of the tapered region. The effect of V-number is expressed as [28]:

(3) $$V_{t}=\frac{2\pi r_{t}\left(z\right)}{\lambda }\sqrt{n_{co}-n_{en}}$$

where Z is the coordinate along the fiber and the radius of the tapered fiber, $r_{t}(z)$ is a function of Z, and nen is the refractive index (RI) of the external environment. When the RI of the environment changes, the value of the V-number will be changed, causing the change in the output power.

**Figure 1:**
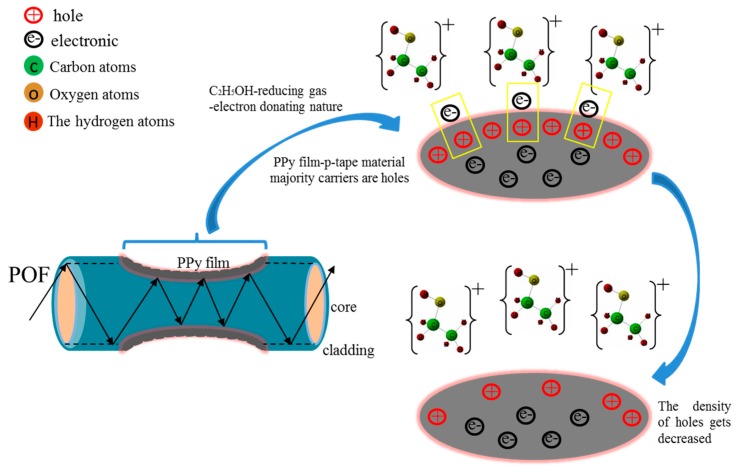
The schematic diagram of the proposed sensor.

The properties of the PPy film vary when in contact with ethanol due to environmental conditions. The schematic diagram of the proposed sensor is shown in Figure 1. PPy represents a p-type material and when it interacts with the reducing gas, such as ethanol ($C_{2}H_{5}\mathrm{OH}$), there is a reduction in the majority carrier (hole) density due to electron-donating nature of $C_{2}H_{5}\mathrm{OH}$ which results in a decrease in the conductivity of the PPy film [30]. On the other hand, the π-conjugated system of the PPy film and the $C_{2}H_{5}\mathrm{OH}$ doping process leads to polaron and bipolaron states [25]. The doped molecules take the π-conjugated electrons, causing the π-conjugated electron rearrangement and polarization [31]. With the increase in the doping concentration, the density of the internal polaron and bipolaron of the PPy molecule also increases, and a new transition level in the visible range is generated. Therefore, the doping process causes the PPy absorbance to increase in the visible light region, resulting in a decrease in the output power [32,33].

## 3. Experimental Evaluation

### 3.1. Fabrication of POF-PPy Probes

The pyrrole (CP100ml, Sinopharm Chemical Reagent Co., Ltd) was stored in a brown bottle and protected from the light. The FeCl_3_ (CP500g, Sinopharm Chemical Reagent Co., Ltd) was stored in a sealed container. All of the used chemicals were analytical-grade reagents and they were used without the need for extra processing.

**Figure 2:**
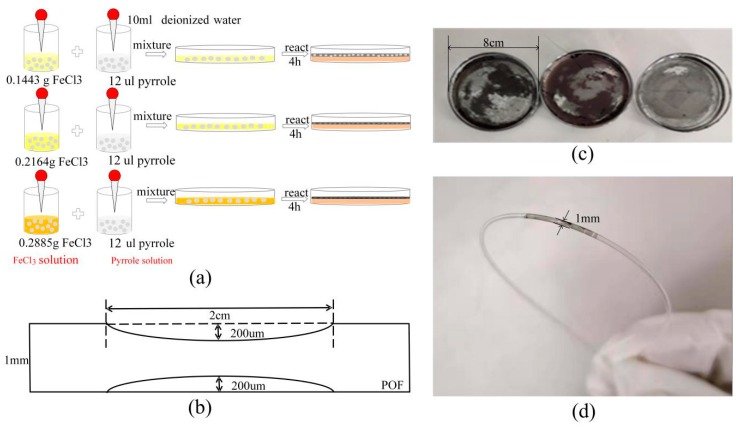
(**a**) The schematic diagram of the preparation of the polypyrrole (PPy) films. (**b**) Parameters of the roughened plastic optical fiber (POF) taper. (**c**) The PPy films at different FeCl_3_ to pyrrole ratios; from right to left, n$\mathrm{Fe}{\mathrm{Cl}}_{3}$: n_pyrrole_ = 5:1, 15:2, and 10:1. (**d**) The POF-PPy1 sensor probe.

The three FeCl_3_ powders having the weight of 0.1443 g, 0.2164 g, and 0.2885 g were used. A pipette took three pyrrole resolutions of 12 μL. As shown in Figure 2a, 10 mL of deionized water was added to all of the three FeCl_3_ powders, respectively, and they were stirred evenly in the beaker. The pyrrole solutions were disposed of in the same manner as the FeCl_3_ solutions. The three FeCl_3_ solutions and pyrrole solutions were poured into three glass-culture dishes with a diameter of 8 cm and left to react at room temperature for 4 h. The three black-gray PPy films were formed on the surface, as shown in Figure 2c. In order to keep the shape of the PPy film, a needle was used to extract the reaction solution. After that, the PPy films were rinsed with the deionized water to remove the residual ionic impurities and unresponsive solution from the surface.

A POF (Mitsubishi, Tokyo, Japan, SK40) with a length of 30 cm was used, which had a core of 980 μm and a fluorinated-polymer cladding of 10 μm. It was polished using 2000 mesh sandpaper at a distance of 2 cm in the middle of the fiber. The electron microscope controlled the processing depth. The roughened POF taper was cleaned with deionized water. The parameters of the fiber are presented in Figure 2b. The 20 mm × 4 mm rectangular mold cut the PPy films for subsequent use. Dip-coating was used to modify the PPy film on the fiber surface. The roughened POF taper was inserted under the film and slowly taken out at room temperature and dried at room temperature (RT) for 24 h. The POF-PPy1 probe is shown in Figure 2d.

### 3.2. Ethanol Sensing Measurements

**Figure 3:**
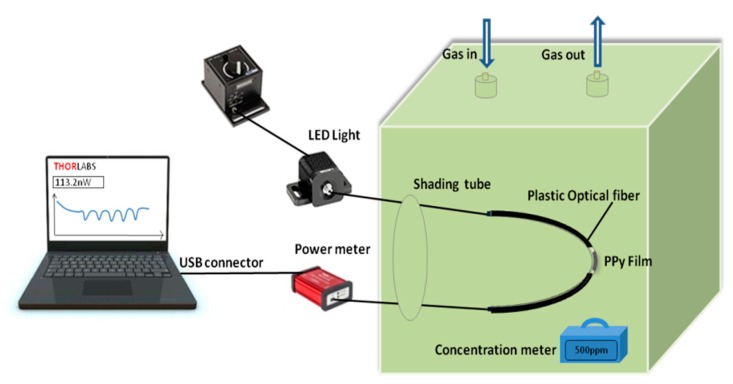
The experimental setup of the POF-PPy sensor.

The experimental setup is shown in Figure 3. The POF-PPy probe was put in the gas chamber with the dimensions of 200 mm × 100 mm × 100 mm. Titration was used to construct the experimental environments of different concentrations of ethanol gas. The concentration of the ethanol gas was calculated according to the amount of the ethanol solution injected and the volume of the gas chamber. The fiber was connected to a 660 nm fiber-coupled LED light source (M660F1, Thorlabs, Newston, NJ, USA) and the other side was connected to the power meter (PM100USB, Thorlabs, Newston, NJ, USA). The change in the output power was displayed on the computer screen. The concentration meter in the chamber (SmartPro10, Sundo, China) monitored the concentration of the ethanol gas chamber in real-time to test the sensitivity and detection range. The accuracy of the concentration meter was 0.01 ppm.

## 4. Results and Discussion

### 4.1. Morphology

#### 4.1.1. Fibers

**Figure 4:**
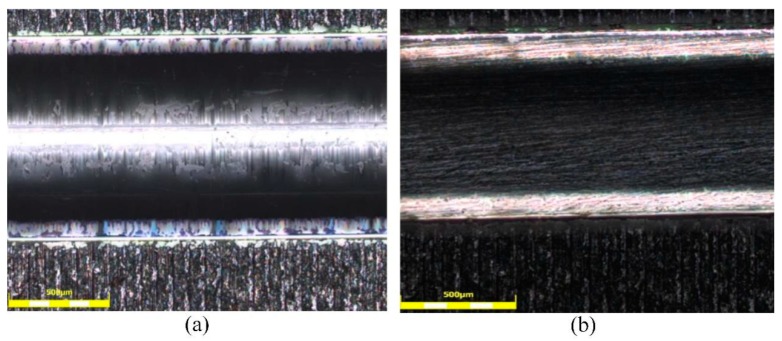
Microscope image of (**a**) unprocessed POF and (**b**) roughened POF.

It can be seen from Figure 2d that the prepared sensor is stable in form. To further investigate the surface of the fiber structure, the micrographs of the unprocessed and roughened POFs presented under the 500-μm scale are shown in Figure 4. In Figure 4a, it can be seen that the surface of the unprocessed POF was smooth, but there were some scratches. The surface of the roughened POF was striped and the stripes had a different depth, as shown in Figure 4b. The roughened POF gave an increase in the region of interaction and could be better attached to the PPy film owing to the higher adhesion than the unprocessed POF.

#### 4.1.2. PPy Films

**Figure 5:**
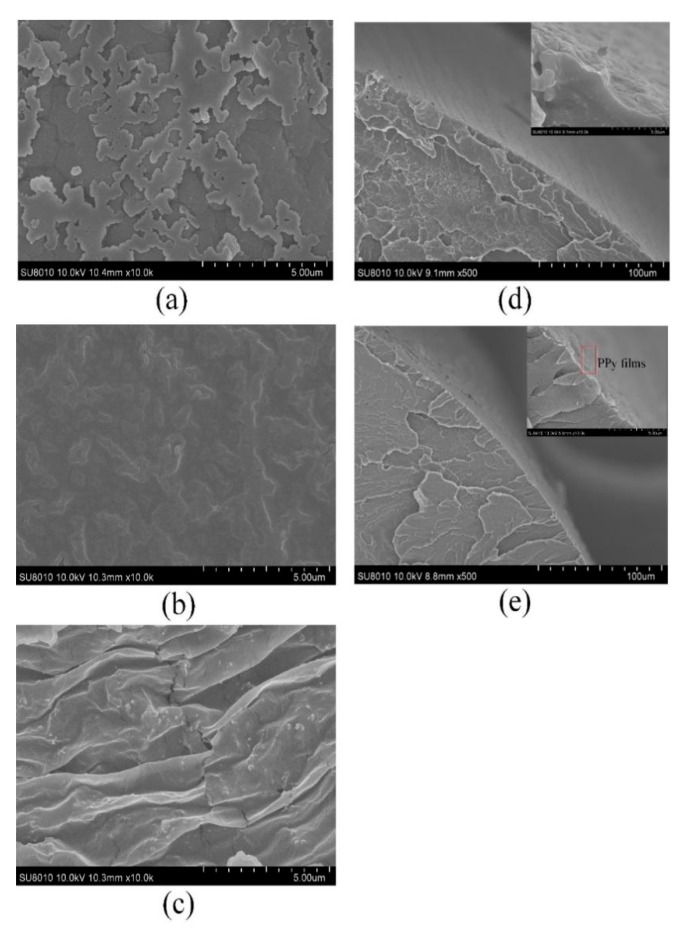
Micrographs of PPy films: (**a**) PPy1 (n$\mathrm{Fe}{\mathrm{Cl}}_{3}$:n_pyrrole_ = 5:1), (**b**) PPy2 (n$\mathrm{Fe}{\mathrm{Cl}}_{3}$:n_pyrrole_ = 15:2), (**c**) PPy3 (n$\mathrm{Fe}{\mathrm{Cl}}_{3}$: n_pyrrole_ = 10:1), (**d**) roughened POF taper, (**e**) roughened POF taper with PPy2 film.

The micrographs of the three PPy films (PPy1, PPy2, and PPy3) observed under the 5-μm scale are shown in Figure 5a–c, respectively. As shown in Figure 5a, due to insufficient pyrrole content, the resulting PPy1 film was not uniform and its thickness was approximately 20 µm. Compared with the PPy1 film, the PPy2 film had a stable morphology and smooth surface, as shown in Figure 5b. According to microscope observation, the film thickness was about 112 µm. Further, as shown in Figure 5c, the wrinkles increased and the PPy3 film was prone to cracks, having a thickness of about 132 µm. It can be seen in Figure 5d that there was a rugged and uneven surface of the roughened POF taper. The picture in the upper right corner of Figure 5d showed the rough surface more clearly. Lastly, Figure 5e shows that the roughened POF taper and the PPy2 film were closely attached.

### 4.2. Response Behavior of POF-PPy Sensor

#### 4.2.1. Reactant Concentration

**Figure 6:**
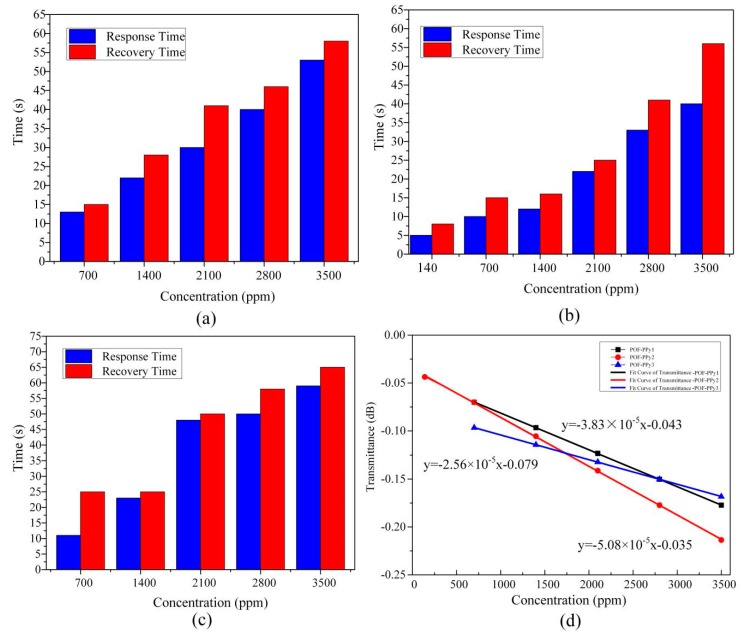
Response and recovery times of: (**a**) POF-PPy1, (**b**) POF-PPy2, (**c**) POF-PPy3, and (**d**) the sensitivity of the proposed sensor.

In order to study the effect of three reactant concentrations on the gas sensitivity of the prepared PPy films, the response and recovery time of the ethanol gas in the range of 0–3500 ppm of the three sensitive probes were measured at room temperature (RT). The response and recovery time of the proposed sensor were considered as time passed until the responses reached 99% of their respective values. The change in power was determined by the input power and output power reaching the gas detection value. The response time, recovery time, and power variation of the three sensitive probes at different ethanol concentrations are presented in Figure 6. As presented in Figure 6a–c, the blue column represents the response time, and the red one represents the recovery time. At the same ethanol concentration, the response and recovery times of the POF-PPy2 were the smallest, which proved that POF-PPy2 was more sensitive than the POF-PPy1 and POF-PPy3. In addition, its detection limit was 140 ppm, and the response and recovery times were 5 s and 8 s, respectively. The film of PPy1 was thinner than that of the PPy2, which was damaged after being attached to the POF taper, and a certain degree of crack was formed. The interaction area between PPy1 film and fiber was reduced, which increased the response time and recovery time. In the case of the POF-PPy3, the film was too thick and had many wrinkles. As a result, the adhesion between the film and the optical fiber was small and the PPy3 film easily fell off, resulting in the longest response and recovery times. The sensitivity was represented by the transmittance (dB). The input power was 50 uW and the output power was displayed by the power meter. The sensitivity of the three probes is depicted in Figure 6d, where it can be seen that the POF-PPy2 had the highest sensitivity, which was 5.08 × 10^−5^ dB/ppm.

Comparison of the proposed sensor and the previously reported ones is presented in Table 1. The ethanol gas sensors cited in the Table 1 were tested at room temperature, which was consistent with the experimental conditions in this paper. As mentioned previously, the proposed sensor shows a good response and recovery times when the ethanol gas concentration varies because the PPy is gas-sensitive material with a high sensitivity to the reducing gas. Besides, the proposed sensor has the advantages of simple structure and low cost of PPy films.

#### 4.2.2. Roughened Fiber

A cladding POF taper was prepared using the heat-and-pull technique with a smooth surface; the surface was modified with the PPy2 film. The processing parameters were chosen as shown in Figure 2b. The input power was 50 uW. The response of the cladding POF taper to the ethanol gas was characterized. The experimental of the three probes (POF-PPy1, POF-PPy2, and POF-PPy3) and the cladding POF taper were given in Table 2, where it can be seen that the response of the three roughened POF tapers was much better than that of the cladding POF taper. The reason was that the PPy film was more tightly attached to the roughened POF taper, owing to adhesion and the friction force, which improved sensitivity. In addition, the EW intensity of the roughened POF taper was stronger than that of the cladding POF taper, which had a large influence on the output power and transmittance.

#### 4.2.3. Repeatability

**Figure 7:**
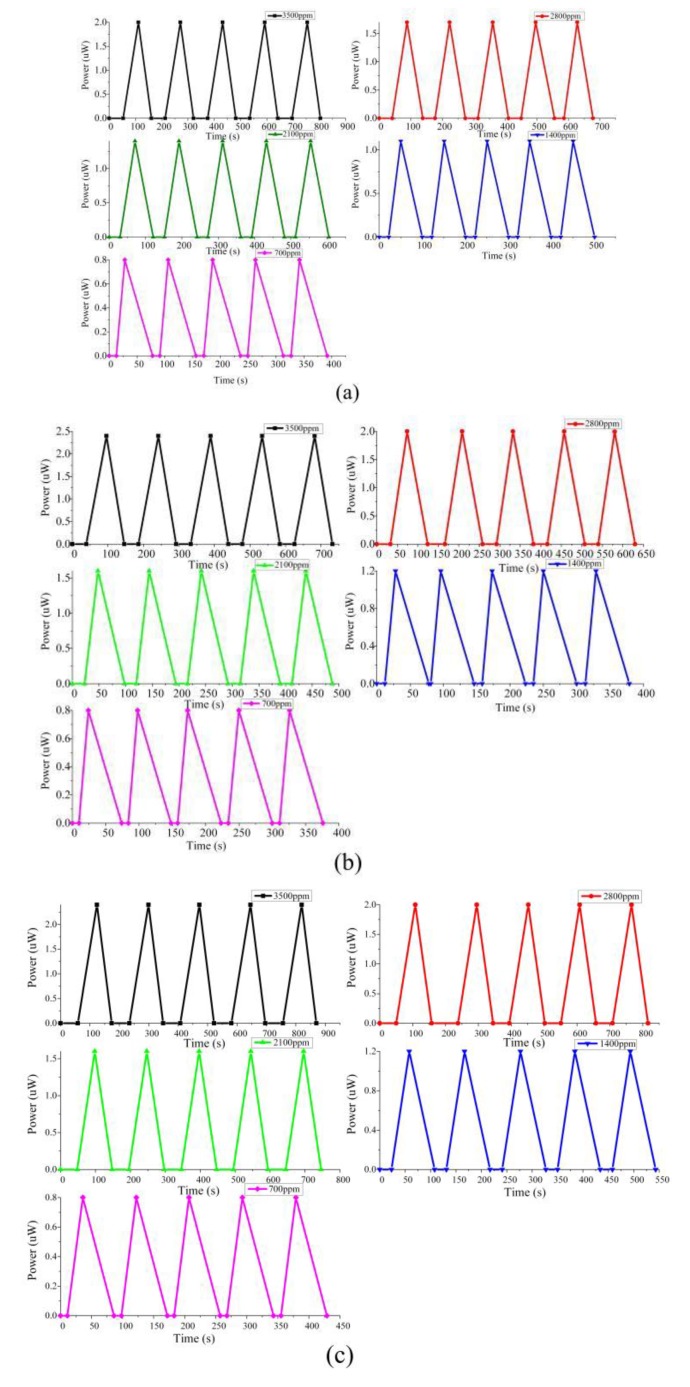
The repeatability results of (**a**) POF-PPy1, (**b**) POF-PPy2, and (**c**) POF-PPy3.

The material properties determine the repeatability of a sensor. In order to evaluate the repeatability of the proposed sensor, three sensitive probes (POF-PPy1, POF-PPy2, and POF-PPy3) were tested. As shown in Figure 3, the connection of the experimental device was completed and five tests were performed on the ethanol gas response of three sensitive probes in the range of 0–3500 ppm at room temperature (RT). The experimental results are shown in Figure 7. Figure 7a–c shows the sensors of POF-PPy1, POF-PPy2, and POF-PPy3, respectively. The five-color curves represent the results of the different ethanol concentrations; five repetitive experiments were performed for each concentration. The results showed that the proposed sensor achieved excellent repeatability in tests, which was the result of the chemical inertness of the PPy film and the adhesion between optical fiber and PPy film. Compared with the repeated experimental results of the cited articles [10,17], it can also be concluded that the chemical properties of the PPy film and the adhesion are two important reasons that affect the repeatability of the proposed sensor.

In addition, it can be seen from Figure 7 that when the concentration was higher (e.g., 3500 ppm), the response time was faster than the recovery time, and when the concentration was lower (e.g., 700 ppm), the recovery time was faster than the response time. The cause of the phenomenon may be that when the gas concentration was large, the concentration of ethanol molecules adsorbed on the polypyrrole (PPy) film was larger, which increased the time during the recovery process. Conversely, when the gas concentration was small, it took time for the gas to flow in the gas chamber due to the influence of the titration process, which would increase the response time. When the gas was pumped out, the recovery time was shorter than the response time.

#### 4.2.4. Bending Radius

**Figure 8:**
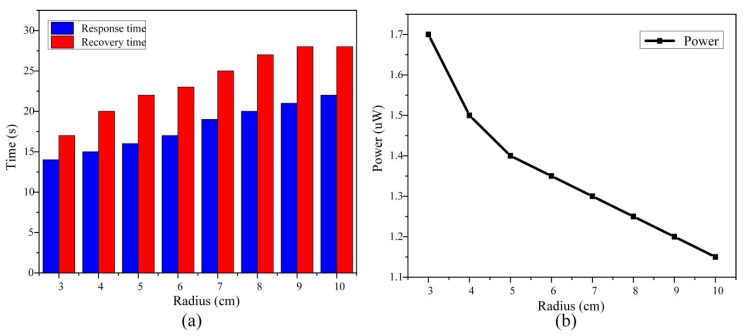
The POF-PPy1 at the bending radius of 3 cm to 10 cm. (**a**) Response and recovery times. (**b**) The output power.

The effect of the bending radius on the gas sensitivity was also measured. The POF-PPy1 was selected and bent it into a U-shaped mold of 3 cm to 10 cm. The effect of the bending radius on the response and recovery times at the gas concentration of 1400 ppm is displayed in Figure 8. It can be seen that when the bending radius was 3 cm, the response was the fastest; namely, the response and recovery times were 14 s and 17 s, respectively. As the bending radius was changing from 10 cm to 3 cm, the response and recovery times gradually reduced and the output power decreased. As a result, the sensitivity of the sensor increased. The main reason was that the macro-bending of the POF taper converts the lower-order modes into the higher-order ones, increasing the power of EW. As the bending radius decreased, the EW and bending loss increased and the output power decreased. Changes in the response and recovery times were related to the penetration depth, and the penetration depth (d) was expressed as [39]:

(4) $$d=\frac{\lambda }{2\pi \sqrt{n_{co}^{2}\sin^{2}\theta c-n_{cl}^{2}}}$$

where θc is the incidence angle of the light at the core-cladding interface. As given in Equation (4), the penetration depth was inversely proportional to the incidence angle of the light. After the fiber was tapered, the penetration depth was approximately given by:

(5) $$d^{\prime }=\frac{\lambda }{2\pi \sqrt{n_{co}^{2}\sin^{2}\theta -n_{en}^{2}}}$$

where $n_{en}$ is the refractive indices of the external environment. θ is the incidence angle of the tapered region of the fiber. After the tapered POF was bent, the transmission angle was reduced. With the other parameters unchanged, the penetration depth ($d^{\prime }$) increased with the decreasing transmission angle. By increasing the penetration depth, the speed of response of the proposed sensor to the ethanol gas also increased. Therefore, as the bending radius decreased, the response time and recovery times also decreased.

## 5. Conclusions

In this work, PPy films were successfully polymerized by the chemical oxide, while the pyrrole content was much less than that of the FeCl_3_ (nFeCl3: n_pyrrole_ is 5:1; 15:2; 10:1). The size of the PPy film is of the micron-order so that it can support the transmission of the light. When the POF taper was in contact with the PPy film, the adhesion helped the PPy film to attach closely to the fiber surface and the friction force of the roughened POF prevents the movement of the PPy film on the surface of the fiber. The proposed sensor was verified experimentally, and it was also compared with the related sensors. The experimental results revealed that the proposed sensor had excellent repeatability and response to the ethanol. The sensitivity to the ethanol was the highest among all the tested sensors, and it was 5.08 × 10^−5^ dB/ppm at the ratio of FeCl_3_ to pyrrole of 15:2. With the decrease of the bending radius of the optical fiber, the response and recovery times were shortened and the output power was increased. The additional advantages of the proposed POF-PPy sensor are that it is easy to fabricate and is inexpensive. In our future work, the PPy films will be applied to the detection of other gases to evaluate the performance of the proposed sensor further.

## Figures and Tables

**Table 1:** Comparison of the proposed sensor and the cited sensors.

Reference	Fiber Structure	Coating Material	Ethanol Gas Detection Range (ppm)	Response and Recovery Times (s)
[34]	clad–modified fiber	nanocrystalline MnCo_2_O_4_	0–500	60, 40 (500 ppm)
[35]	long period grating fiber	ZIF-8	62–666	none
[36]	Silica microfiber	PMMA microwire	0–15,000	250, 9.4
[37]	single-mode silica fiber	nano-sized SnO_2_	1000–20,000	10, 55 (1000 ppm)
[38]	multimode PMMA fiber	polypyrrole/Prussian blue	0–500	none
This paper	POF-PPy2	PPy films	140–3500	5, 8 (140 ppm)

**Table 2:** Comparison between the three POF-PPy sensors and the cladding POF taper sensor with PPy2 film.

Probe	Response Time (s)	Recovery Time (s)	Transmittance (dB)
POF-PPy1	22	28	−0.0966
POF-PPy2	12	16	−0.1055
POF-PPy3	23	25	−0.1144
Cladding POF	55	61	−0.0261
